# Extremely large fractionation of Li isotopes in a chromitite-bearing mantle sequence

**DOI:** 10.1038/srep22370

**Published:** 2016-03-01

**Authors:** Ben-Xun Su, Mei-Fu Zhou, Paul T. Robinson

**Affiliations:** 1 State Key Laboratory of Lithospheric Evolution, Institute of Geology and Geophysics, Chinese Academy of Sciences, P.O. Box 9825, Beijing 10029, China; 2 Department of Earth Sciences, the University of Hong Kong, Pokfulam Road, Hong Kong, China

## Abstract

We report Li isotopic compositions of olivine from the mantle sequence of the Luobusa ophiolite, southern Tibet. The olivine in the Luobusa ophiolite has Li concentrations from ~0.1 to 0.9 ppm and a broad range of δ^7^Li (+14 to −20‰). An inverse correlation of Li concentration and δ^7^Li in olivine from harzburgite suggests recent diffusive ingress of Li into the rock. Olivine from dunite enveloping podiform chromitites shows positive δ^7^Li values higher than those of MORB, whereas olivine from the chromitite has negative δ^7^Li values. Such variations are difficult to reconcile by diffusive fractionation and are thought to record the nature of the magma sources. Our results clearly indicate that the Luobusa chromitites formed from magmas with light Li isotopic compositions and that the dunites are products of melt-rock interaction. The isotopically light magmas originated by partial melting of a subducted slab after high degrees of dehydration and then penetrated the overlying mantle wedge. This study provides evidence for Li isotope heterogeneity in the mantle that resulted from subduction of a recycled oceanic component.

## Introduction

Recycling of oceanic lithosphere, mantle convection and crust-mantle interaction are processes that can produce isotopically heterogeneous mantle. Mantle heterogeneity induced by subduction is well constrained by the Li isotope system, which is sensitive to dehydration and metamorphism. ^7^Li can be released preferentially from subducted slabs resulting in isotopically heavy mantle wedges and light residues^1^,^2^,^3^. Release of Li from the slabs to the mantle wedge is believed to be spatially variable both in amount and isotopic composition^1^,^4^. Heavy Li isotope signatures in OIBs (oceanic island basalts) appear to provide a geochemical tool for identifying recycled inputs into OIB sources^1^,^5^,^6^,^7^,^8^. However, partial melting residues enriched in isotopically light Li have only rarely been reported, but are important for understanding the fate of subducted slabs and the geochemical behavior of Li isotopes.

Light Li isotopic signals have been reported in a few abyssal peridotites and ophiolitic rocks^3^,^9^,^10^. Mantle sequences of ophiolites, particularly those with podiform chromitite deposits, were probably formed originally at mid-ocean ridges and then modified by melt-mantle interaction in suprasubduction zone environments^11^,^12^,^13^. The Luobusa ophiolite in southern Tibet has well-preserved mantle peridotites and podiform chromitites. Most of the podiform chromitites are enclosed in dunite envelopes, which clearly originated by interaction between peridotites and melts^12^,^13^. However, neither the nature nor the origin of the melts has been well constrained. Given that Li isotopes are important for tracing subduction-related processes^2^,^14^,^15^,^16^ and melt-peridotite interaction^17^,^18^,^19^,^20^,^21^,^22^, a ~20-cm-wide reaction zone in the Luobusa peridotite was selected for a detailed Li isotopic study to understand the extent of mantle heterogeneity at a sample scale. *In situ* Li isotopic analyses for olivine from this well-preserved reaction zone revealed dramatic changes of Li isotopic composition across the zone.

### Geological background and petrography

**Figure 1:**
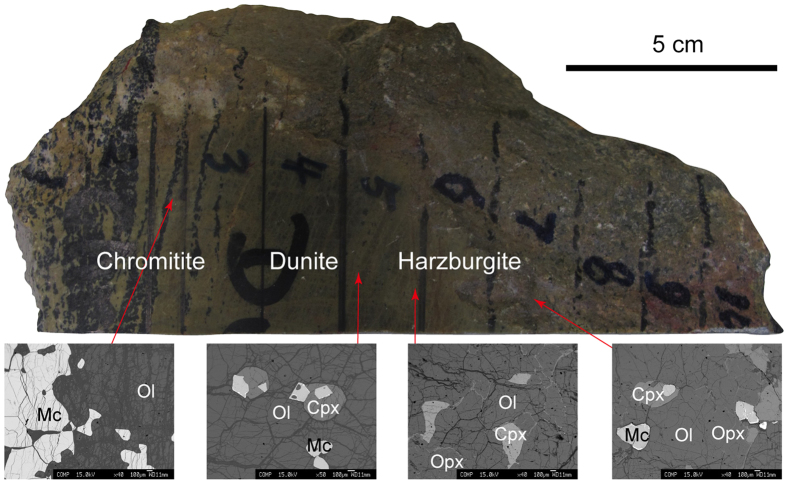
Photograph and back-scattered images of the sample consisting of harzburgite, dunite and chromitite of the Luobusa ophiolite, southern Tibet. Cpx, clinopyroxene; Mc, magnesiochromite; Ol, olivine; Opx, orthopyroxene.

**Figure 2:**
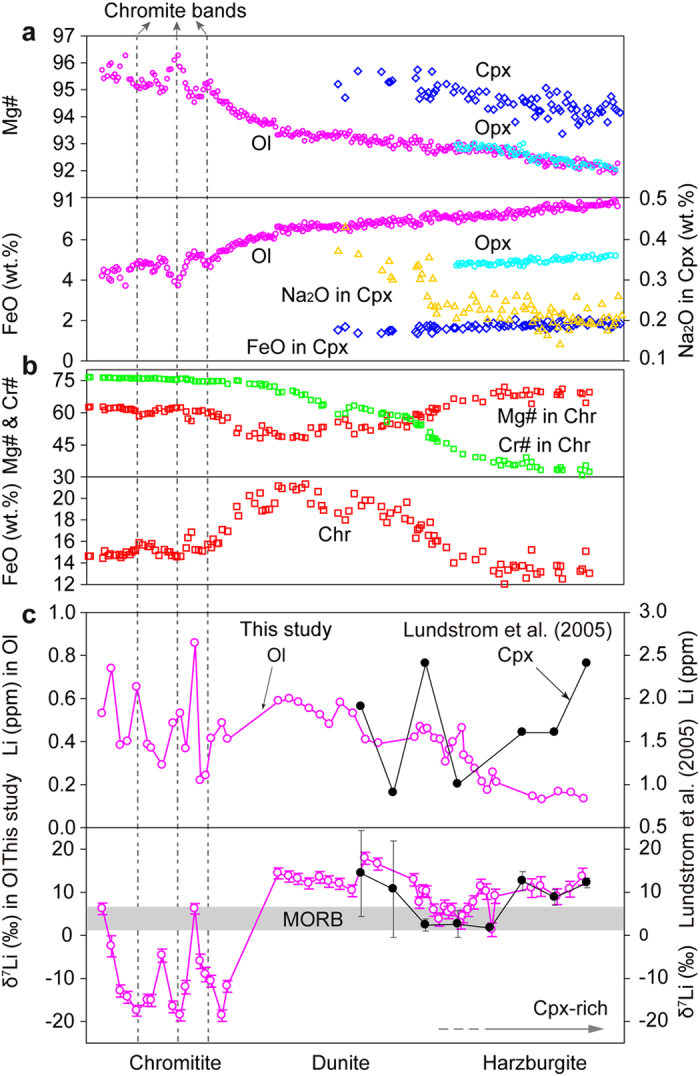
Chemical variations of minerals from harzburgite through dunite to chromitite. (**a**) Mg#, FeO and Na_2_O contents of silicate minerals; (**b**) Mg#, Cr# and FeO contents of magnesiochromite; (**c**) Li isotopic and elemental variations of olivine. A compositional profile of clinopyroxene in lherzolite-harzburgite-dunite transect from the Trinity ophiolite^9^ is also plotted for comparison.

The Tibetan Plateau was formed by the northward accretion of several terranes, separated by sutures, namely from south to north, The Yarlung-Zangbo, Bangong-Nujiang and Kohoxili suture zones. The Cretaceous Luobusa ophiolite lies in the eastern Yarlung Zangbo Suture Zone, about 200 km east-southeast of Lhasa. It contains both mantle and crustal rocks and hosts the largest chromite deposit in China^12^,^23^,^24^. The mantle sequence is composed of harzburgite, dunite and podiform chromitite. The harzburgites in this ophiolite are relatively refractory; all of the silicate minerals have high Mg#s [100 × Mg/(Mg + Fe)] of 92 to 96 and the magnesiochromite has variable Cr#s [100 × Cr/(Cr + Al)] from 30 to 76. The harzburgites also have very low bulk REE concentrations, with HREE ranging from 0.1 to 0.8 × chondrite, MREE from 0.05 to 0.2 × chondrite and LREE from 0.01 to 1.0 × chondrite, although many samples contain 2–3 modal% clinopyroxene^23^. Samples for this study were taken from a 20-cm-wide zone extending from a chromitite band through dunite to harzburgite (Fig. 1). The host harzburgite consists of ~70–75 modal% olivine (Fo_92_), ~20–25% orthopyroxene (Mg# = 92), ~3% clinopyroxene (Mg# = 94) and 1–2% magnesiochromite (Mg# = 70; Cr# = 30). In contrast, the chromitite band consists of 10–50% high-Mg olivine (Fo_95–96_) and 50–95% high-Cr magnesiochromite (Mg# = 57–62; Cr# = 74–76). Olivine is mostly interstitial to the magnesiochromite grains. As seen in Fig. 2, there are regular and systematic variations between the harzburgite and chromitite in mineral abundance and composition. Moving from the harzburgite to the chromitite, the abundance of pyroxene decreases (reaching zero in the dunite), whilst that of olivine increases (Fig. 1; ref. 23). All the silicate minerals show increases in Mg#, with olivine reaching a composition of Fo_95_ at the dunite-chromitite boundary (Fig. 2a). Although the abundance of magnesiochromite remains relatively constant in the harzburgite-chromitite transition zone, its Cr# increases to about 74 at the dunite-chromitite contact, which is relatively sharp (Fig. 2b). The lithologic and chemical characteristics of the studied samples (Figs 1 and 2) are identical to the reported data on harzburgite, dunite and chromitite of the Luobusa ophiolite^12^,^13^,^23^,^24^, indicating that the samples are representative of the mantle sequence as a whole.

### Lithium isotopic compositions of olivine

**Figure 3:**
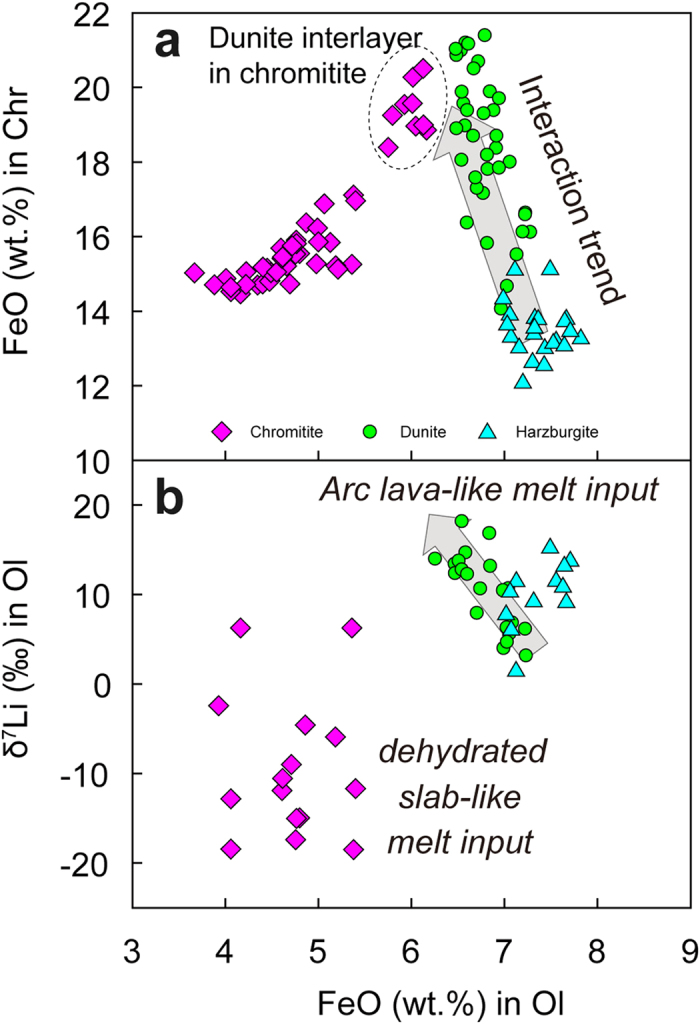
Diagrams of (**a**) FeO in olivine vs. FeO in magnesiochromite and (**b**) FeO in olivine vs. δ^7^Li in olivine showing interaction trend from harzburgite to dunite and Li isotopic variations in the formation of dunite and chromitite.

All of the olivine grains analyzed in this study, regardless of their host lithology, have low Li contents (~0.1 to 0.9 ppm). Olivine in the harzburgite generally has lower concentrations (0.13 to 0.35 ppm) than olivine in the dunite (0.30 to 0.60 ppm), however the concentration in the dunite drops markedly to 0.22 ppm at the contact with the chromitite band (Table S1; Fig. 2c). The δ^7^Li values of olivine decrease from +13.6‰ in the Cpx-bearing harzburgite to +2.9‰ in the Cpx-poor harzburgite, and then increase again immediately adjacent to the dunite, only to drop steeply adjacent to the dunite-chromitite contact (Table S1; Fig. 2c). Olivine within the chromitite has variable Li abundances (0.20 to 0.90 ppm) and extreme isotopic heterogeneity, ranging from very light (-20‰) to MORB values (+7‰) (Fig. 2c). These olivine grains have δ^7^Li values lower than those in the dunite zone and in the interlayered dunite within the chromitite. These variations correlate with variations in the Mg#s and FeO contents of both olivine and magnesiochromite (Figs 2 and 3).

## Discussion

### Primary features of Li isotopes

**Figure 4:**
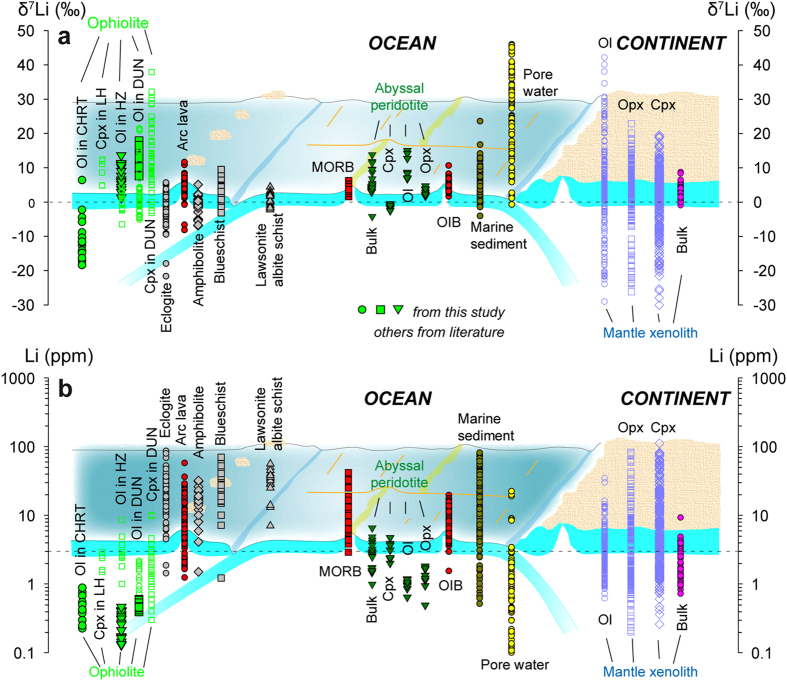
Li isotopic (**a**) and elemental (**b**) compositions of the main hosts and mineral separates in oceanic and continental settings. CHRT, chromitite; DUN, dunite; HZ, harzburgite; LZ, lherzolite. Marine sediment, refs 25, 26, 44 and 60; Pore water, ref. 25, 27 and 28; Arc lava, refs 1, 42, 43, 44, 45, 46 and 61; Abyssal peridotite, refs 3 and 10; Ophiolite, unfilled symbols from ref. 9 and filled symbols from this study; MORB, refs 6, 49 and 51; OIB, refs 7, 44, 50 and 52; Lawsonite albite schist, refs 39, 40, 41; Blueschist, refs 15, 39 and 40; Amphibolite, ref. 39; Eclogite, refs 2, 15 and 41; Bulk of peridotite xenolith, refs 18, 19, 32, 53, 54, 55, 56, 57; Olivine, orthopyroxene and clinopyroxene of peridotite xenoliths, refs 4, 21 and 22 and references therein.

Because sediment pore water has variable and overall high δ^7^Li values (0.0 to +46‰), fluid-rock interaction involving these media should enrich heavy Li isotope signatures of the rocks (Fig. 4a,b; refs 14, 25, 26, 27, 28, 29, 30). Thus, this medium cannot account for the extremely low δ^7^Li values of olivine in the Luobusa chromitite. Serpentinization removes Li, preferentially ^6^Li, from the mineral grains to form Li-rich serpentine with low δ^7^Li 26,31. Thus, the involvement of serpentine from intergranular spaces and microcracks could potentially produce analyses with increased Li concentration but with decreased δ^7^Li^9^,^31^, unlike the decrease in Li concentration observed in the magnesiochromite bands, where microcracks are relatively well developed (Figs 1 and 2). High-temperature equilibrium fractionation of Li isotopes between melt and mantle peridotite is minor (<0.5‰) and produces covariations of δ^7^Li and Li abundance^32^,^33^, which are inconsistent with our results.

Kinetic processes represent another possible means of altering Li isotopes. Diffusion-driven fractionation of Li isotopes generally assumes ingress of Li from an external source into rocks or minerals, such as diffusion of Li from Li-rich pegmatite into country rocks^34^, or from olivine into coexisting clinopyroxene during cooling of peridotites^32^,^35^. In the case of Li diffusion from melts to wall-rock peridotites^9^, the melts become progressively depleted in ^6^Li because its diffusion rate is more rapid than ^7^Li^31^, leading to high-δ^7^Li melts and low-δ^7^Li wall-rock peridotites. This mechanism could potentially account for the observed decreases of δ^7^Li with increasing Li abundance in olivine from the Luobusa harzburgite and clinopyroxene from the Trinity harzburgite^9^ (Fig. 2c), suggesting diffusive ingress of Li into the harzburgite. However, such model does not adequately explain the observed isotopically light olivine with low Li abundance in the chromitite band and the ^7^Li-rich olivine and high Li abundance of olivine in the dunite (Figs 2c and 3b). Studies by Dohmen *et al.*^36^ and Ritcher *et al.*^37^ revealed a complex diffusion behavior of Li and they accordingly proposed a coupled fast and slow diffusion mechanism. The fast mechanism of Li diffusion is unlikely to be dominant in most natural systems; under slow diffusion, the Li diffusion rate is about an order of magnitude faster than diffusion of Fe, Mg and many other divalent cations in olivine. Therefore, diffusion rate and cation exchange are relevant to the element content in the minerals. The overall decreasing FeO content and increasing Mg# in minerals, which show no correlations with Li variations in olivine in the harzburgite-dunite-chromitite profile (Fig. 2), could not have resulted solely from diffusion.

Thermal gradients can also fractionate Li isotopes resulting in ^7^Li enrichment at the low-temperature end^38^. The harzburgite, occurring as wall rock of dunite in the Luobusa ophiolite^12^,^23^, should have had a lower temperature than the melts from which the dunite formed. The contact between Cpx-poor harzburgite and dunite, however, has low δ^7^Li signatures (Fig. 2c), contrary to what would be expected from thermal isotope fractionation.

Therefore, we conclude that the olivine in the Luobusa chromitite preserves primary Li abundances and isotopic compositions. Given that the mineral assemblage in the chromitite band consists only of olivine + magnesiochromite, and that the magnesiochromite structurally contains minor or no Li, we assume that the Li contents and isotopic values in olivine of the Luobusa dunite and chromitite are representative of the whole rock samples and thus that the observed Li isotope heterogeneity in these samples reflects their mantle sources.

### Constraints on the formation of dunite

**Figure 5:**
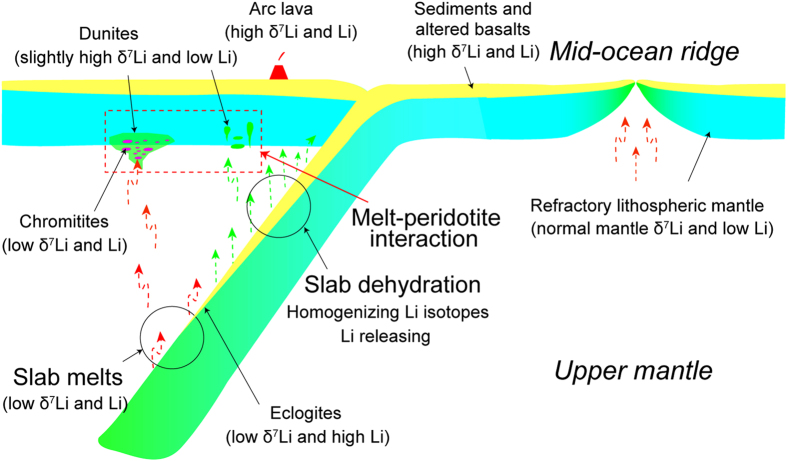
A cartoon showing variations of Li and its isotopes and formation of dunite and chromitite in an intra-oceanic subduction zone. The δ^7^Li values and Li concentrations are relative to normal mantle values of δ^7^Li = +2 to +6‰ and Li = 1 to 2 ppm (refs 6,22,49). See exact ranges in Fig. 4. Although the formation of dunite and chromitite is treated as two processes in this model, we suggest that both are formed in a single, continuous process.

Compositional changes of Li isotopes in olivine across the reaction zone are compatible with changes in FeO contents in the olivine and magnesiochromite (Figs 2 and 3) and Na_2_O contents in the clinopyroxene (Fig. 2a). Similarly, there are also gradual changes in the whole-rock compositions, i.e. increasing LREE and IPGE and decreasing HREE and PPGE away from the harzburgite, accompanied by abrupt changes at the harzburgite-chromitite contact^23^. Similar Li isotopic variations in clinopyroxene were also reported along a lherzolite-harzburgite-dunite profile from the Trinity ophiolite, USA (Fig. 2c; ref. 9). This pattern is consistent with a reaction process whereby Li isotopes are fractionated during diffusion from melts in conduits to the surrounding peridotites. Such a reaction considerably raises the Li isotope ratios but has little influence on the absolute or relative abundances of the element in olivine and clinopyroxene (Figs 2b and 5; ref. 9). Bulk rock and mineral separates of ophiolitic peridotites and abyssal peridotites have low Li concentrations of <3 ppm, less than MORBs and OIBs (Fig. 4b), which can be attributed to melt extraction prior to peridotite-melt interaction. Therefore, Li isotopic variations in the harzburgite require a combined process of melt extraction and diffusion, which should also be responsible for the petrologic observation that clinopyroxene extends much farther toward the chromitite than orthopyroxene, because of the preferential dissolution of clinopyroxene during melting and preferential formation of clinopyroxene during metasomatism (Fig. 2a). The elevated δ^7^Li values with an average of 10.1‰ in the dunite indicate that the heavy Li isotope signature was generated from a melt with a Li isotope composition falling in the range of arc lavas (δ^7^Li = −8.4 to 11.4‰) (Fig. 4a). In the chromitite from the Luobusa ophiolite, olivine crystals in the interlayered dunite bands have the highest δ^7^Li (Fig. 2c) and probably co-crystallized with those in the dunite, but they were subsequently modified by melts with light Li isotope signatures from which the magnesiochromite crystallized. Furthermore, the contrasting features of Li elemental and isotopic values in olivine from the chromitite suggest that its origin was different from that of the dunite.

### Linking light δ^7^Li of olivine in chromitite with a dehydrated slab

Rocks formed by prograde metamorphism undergo variable degrees of dehydration and generally display decreasing trends in δ^7^Li values and Li abundances (Fig. 4), in the following order; lawsonite/albite schists (average Li = 30.8 ppm; δ^7^Li = 0.93‰), blueschists (average Li = 28.6 ppm; δ^7^Li = 2.36‰), amphibolites (average Li = 15.7 ppm; δ^7^Li = −0.89‰) and eclogites (average Li = 22.6 ppm; δ^7^Li = −1.49‰). On one hand, the Li isotopic variations in these metamorphic rocks are in very good agreement with the geochemical behavior of Li isotopes in subducted slabs during dehydration. That is, isotopically heavy Li is released into the mantle wedge in subduction zones, whereas the isotopically light component is subducted into the deeper mantle (Fig. 4; ref. 2). On the other hand, high Li concentrations in these metamorphic rocks are incompatible with expected Li behavior during dehydration because the process is expected to cause significant removal of Li from the rocks due to its moderately incompatible and fluid-affinity nature. Elevated Li concentrations and isotopic values in some of these rocks have been interpreted as resulting from Li addition from an aqueous fluid during exhumation and/or retrograde processes, because the rocks are presumed to have originally contained much lower Li concentrations and δ^7^Li values under mantle conditions^2^,^15^,^39^,^40^,^41^. The heavy Li isotope released from the slabs probably contributes to the composition of arc lavas, whereas the isotopically light Li slab residues are subducted deep into the mantle^1^,^29^,^42^,^43^,^44^,^45^,^46^, where they may form distinct reservoirs^2^,^4^. Light Li isotope melts have been observed in highly metasomatized mantle xenoliths that may have been derived from such reservoirs^4^,^21^,^22^.

It is believed that the chromitites in Luobusa were precipitated from hydrous, high-Mg magmas undergoing differentiation (Fig. 3; refs 12 and 47). If the differentiation of the mafic magma was not accompanied by Li isotope fractionation^48^, the large Li isotopic variation in olivine from the chromitites cannot be explained by this process. The low δ^7^Li values, with an average of −10.2‰, and the low Li concentrations in olivine from the Luobusa chromitites (Figs 3, 4 and 5) are incompatible with dehydrated fluids from a subducted slab or an asthenospheric magma (e.g., MORB) but are most likely related to melting of a dehydrated slab^1^,^42^,^43^,^44^,^45^,^46^. The tearing and breakoff of the subducted slab, possibly along the transitional contact between amphibolites and eclogites, and subsequent asthenospheric upwelling probably caused partial melting of the dehydrated slab^13^.

Different degrees of mixing between asthenospheric melts and slab melts can explain the large variations in Li concentrations and δ^7^Li values. The involvement of a dehydrated slab should result in production of siliceous and oxidized melts that rapidly trigger magnesiochromite crystallization^12^,^13^. Magnesiochromite grains are suspended in upward-moving melts as they migrate through the overlying mantle wedge. Such melts eventually deposit magnesiochromite in magma conduits in the uppermost mantle. The formation of dunite and chromitite should be a continuous, linked process to account for the close affinity of dunite and chromitite in most ophiolites^13^.

### Implications for heterogeneous Li isotopes in the mantle

Studies of MORB lavas have revealed that they are derived from a relatively depleted mantle reservoir with a Li isotopic composition of δ^7^Li = +1.6 to +6.2‰ (Fig. 4a; refs 6 and 49). Large-scale heterogeneity of the mantle has been documented in OIB lavas (+1.4 to +10.4‰; Fig. 4a; refs 7, 50, 51, 52) and mantle xenoliths (−1 to +10‰; refs 18, 19, 32, 53, 54, 55, 56, 57). Compared to MORB and OIB lavas, fresh oceanic peridotites (bulk: δ^7^Li = −4.2 to + 13.8‰; refs 3 and 10) and those from ophiolites (i.e. olivine in dunite and chromitite) have much larger Li isotopic variations (δ^7^Li = −18.6 to +21.3‰) (ref. 9 and this study). Likewise, mantle xenoliths display ~60‰ δ^7^Li variation among their constituent minerals (Fig. 4a), although much of the variability is poorly constrained, indicating highly variable Li isotopes in xenoliths. These data indicate that the lithospheric mantle is more heterogeneous in Li isotopes and preserves more compositional signatures from slab melts than the asthenospheric mantle, and that Li isotopes are not necessarily equilibrated at mantle temperatures as previously expected^32^,^35^.

In a global perspective, oceanic crust recycled into the mantle via subduction could be partly returned to the Earth’s surface by means of dehydration, magmatism and exhumation. However, subducted slabs and their stagnant fragments in the mantle are far less voluminous than the oceanic crust that once existed^58^. Most of the fragments were probably successively involved in melt-peridotite interaction in oceanic subduction zones to facilitate the large volumes of melt needed for the modification of the oceanic mantle and occasional formation of podiform chromitite deposits^13^. Because dehydrated slabs would be depleted in Li due to dehydration, their light isotopic signature might be easily overprinted upon reaction with peridotites.

## Conclusions

The Luobusa ophiolite preserves Li isotopic variations produced both during formation at mid-ocean ridges and by subsequent modification in a suprasubduction zone setting. Olivine from the harzburgite shows negative co-variation between Li concentrations and δ^7^Li values due to diffusive ingress of Li from the melts. The Li isotopic features of the dunite are comparable to arc-like melts. The melts from which the magnesiochromite crystallized are inferred to have been depleted in Li content and enriched in ^6^Li relative to ^7^Li, thus producing values inferred for highly dehydrated slabs. These two types of melts continuously and progressively reacted with the oceanic lithospheric mantle, resulting in the formation of dunite and chromitite, and accounting for the observed Li isotope heterogeneity.

## Methods

### Major element analysis

Major element compositions of minerals were determined by wavelength dispersive spectrometry using JEOL JXA8100 electron probe microanalyzer (EPMA) at the Institute of Geology and Geophysics, Chinese Academy of Sciences (IGGCAS), Beijing, China. The EPMA analyses were carried out at an accelerating voltage of 15 kV and 10 nA beam current, 5 μm beam spot and 10–30 s counting time on peak. Natural and synthetic minerals were used for standard calibration. A program based on the ZAF procedure was used for matrix corrections. Typical analytical uncertainty for all of the elements analyzed is better than 1.5%.

### Li isotope analysis

**Figure 6:**
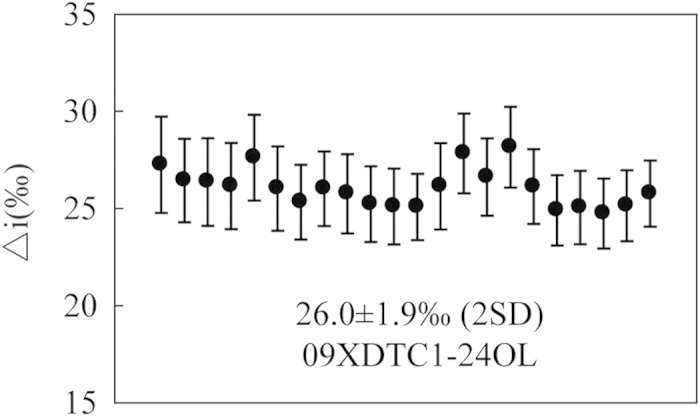
Standard Li isotopic variation throughout the analyses with 2σ error bars.

*In situ* Li isotope measurements of olivine on thin sections were performed on Cameca IMS-1280 SIMS at IGGCAS. The O^−^ primary ion beam was accelerated at 13 kV, with an intensity of about 15 to 30 nA. The elliptical spot was approximately 20 × 30 μm in size. Positive secondary ions were measured on an ion multiplier in pulse counting mode, with a mass resolution (M/DM) of 1500 and an energy slit open at 40 eV without any energy offset. A 180-second pre-sputtering without raster was applied before analysis. The secondary ion beam position in the contrast aperture, as well as the magnetic field and the energy offset, was automatically centred before each measurement. Thirty cycles were measured with counting times of 12, 4 and 4 seconds for ^6^Li, background at the 6.5 mass, and ^7^Li, respectively. Olivine sample 09XDTC1-24OL with Fo of 94.2 was used as standard^59^ and its similar composition to the analyzed olivine eliminates any possible contribution of matrix to the observed data. The measured δ^7^Li values are given as δ^7^Li ([(^7^Li/^6^Li)_sample_/(^7^Li/^6^Li)_L-SVEC_−1] × 1000) relative to units of the standard NIST SRM 8545 (L-SVEC). The instrumental mass fractionation is expressed in δ^7^Li = δ^7^Li_SIMS_−δ^7^Li_MC-ICPMS_. Twenty two analyses on the standard in the study yielded homogeneous Li isotopic composition with Δi = 26.0 ± 1.9‰ (2SD) (Fig. 6). Matrix effect, of which δ^7^Li increased by 1.0‰ for each mole percent decrease in forsterite component of olivine^59^, was considered for calibration. The external 2σ errors of the isotope compositions for both the standards and the samples are less than 2.5‰.

## Additional Information

**How to cite this article**: Su, B.-X. *et al.* Extremely large fractionation of Li isotopes in a chromitite-bearing mantle sequence. *Sci. Rep.* **6**, 22370; doi: 10.1038/srep22370 (2016).

## Supplementary Material

Supplementary Dataset 1
